# Treatment of atypical hemolytic uremic syndrome with eculizumab in a patient presenting with neuropsychiatric prodrome: a case report

**DOI:** 10.3389/fimmu.2025.1542973

**Published:** 2025-04-08

**Authors:** Hanze Yang, Jingdong Zhang, Hao Wu, Hongzhao Xu, Zhonggao Xu

**Affiliations:** Department of Nephrology, The First Hospital of Jilin University, Changchun, Jilin, China

**Keywords:** atypical hemolytic uremic syndrome, thrombotic microangiopathy, eculizumab, neuropsychiatric prodrome, intermittent seizures, posterior reversible encephalopathy syndrome

## Abstract

Atypical hemolytic uremic syndrome (aHUS) is a rare thrombotic microangiopathy (TMA) caused by dysregulation of the complement system. It is characterized by microangiopathic hemolytic anemia, thrombocytopenia, and acute kidney injury. Owing to its diverse and nonspecific clinical manifestations, early diagnosis of the condition is challenging and typically requires excluding other TMA-related conditions, such as thrombotic thrombocytopenic purpura and hemolytic uremic syndrome caused by *Escherichia coli* infection. Accurate diagnosis relies on the recognition of typical TMA symptoms, laboratory testing, and the exclusion of other conditions. Treatments typically include plasma exchange, supportive care, and complement-targeted therapy. Eculizumab, a complement component 5 inhibitor, plays a crucial role in aHUS treatment in severe cases as well as when traditional interventions fail. In this case report, we described a female Han Chinese patient who developed aHUS following an upper respiratory tract infection, initially presented with intermittent seizures, and received treatment with eculizumab, plasma exchange, and hemodialysis. The patient ultimately remained dialysis-dependent; however, they achieved complete remission for other systemic complications of aHUS. We emphasized in this case report the importance of timely diagnosis and treatment of aHUS as well as the potential value of eculizumab in improving patient outcomes. Furthermore, successful treatment and follow-up results provide insights into the management of this rare disease, including long-term dialysis requirements and disease monitoring after remission. Thus, clinicians can better understand the clinical manifestations of aHUS and its associated diagnostic challenges, treatment strategies, and long-term management needs.

## 1 Introduction

Atypical hemolytic uremic syndrome (aHUS) is a severe thrombotic microangiopathy (TMA) characterized by the triad of thrombocytopenia, microangiopathic hemolytic anemia, and acute kidney injury. Without timely treatment, most patients rapidly progress to end-stage renal disease (1). Eculizumab, an anti-complement 5 (C5) therapy, can significantly improve aHUS outcomes and reduce the risk of progression to stage 5 chronic kidney disease from 60–70% to 10–15% (2). Notably, the etiologies of aHUS are complex and diverse with no clear diagnostic criteria. Therefore, some patients miss the optimal treatment window, exacerbating disease-related damage throughout the body.

We report the case of a Han Chinese female patient who developed intermittent seizures following an upper respiratory tract infection and presented with hemolytic anemia, thrombocytopenia, and acute kidney injury. She was initially diagnosed with encephalitis and was later confirmed to have aHUS after excluding thrombotic thrombocytopenic purpura (TTP) and Shiga toxin-producing *Escherichia coli*-associated hemolytic uremic syndrome (STEC-HUS). The patient was treated with eculizumab, plasma exchange, and hemodialysis.

## 2 Case description

**Figure 1:**
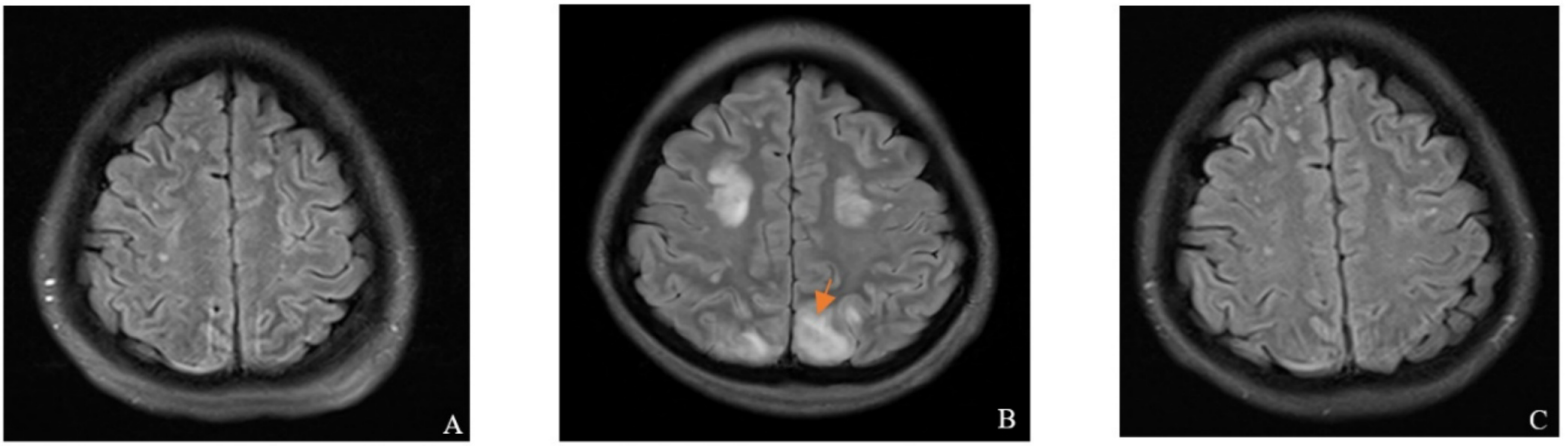
Brain MRI findings of the patient. **(A)** On admission, the MRI showed multiple abnormal signals in the brain, and microvascular thrombosis could not be ruled out. **(B)** Ten days after admission, the MRI revealed multiple abnormal signals in the brain (Where the orange scissors point), with a high likelihood of PRES. **(C)** After four weeks of eculizumab treatment, the MRI indicated multiple abnormal signals in the brain, which were strongly suggestive of PRES; however, the lesions showed improvement compared with the previous MRI results. MRI, magnetic resonance imaging; PRES, posterior reversible encephalopathy syndrome.

**Figure 2:**
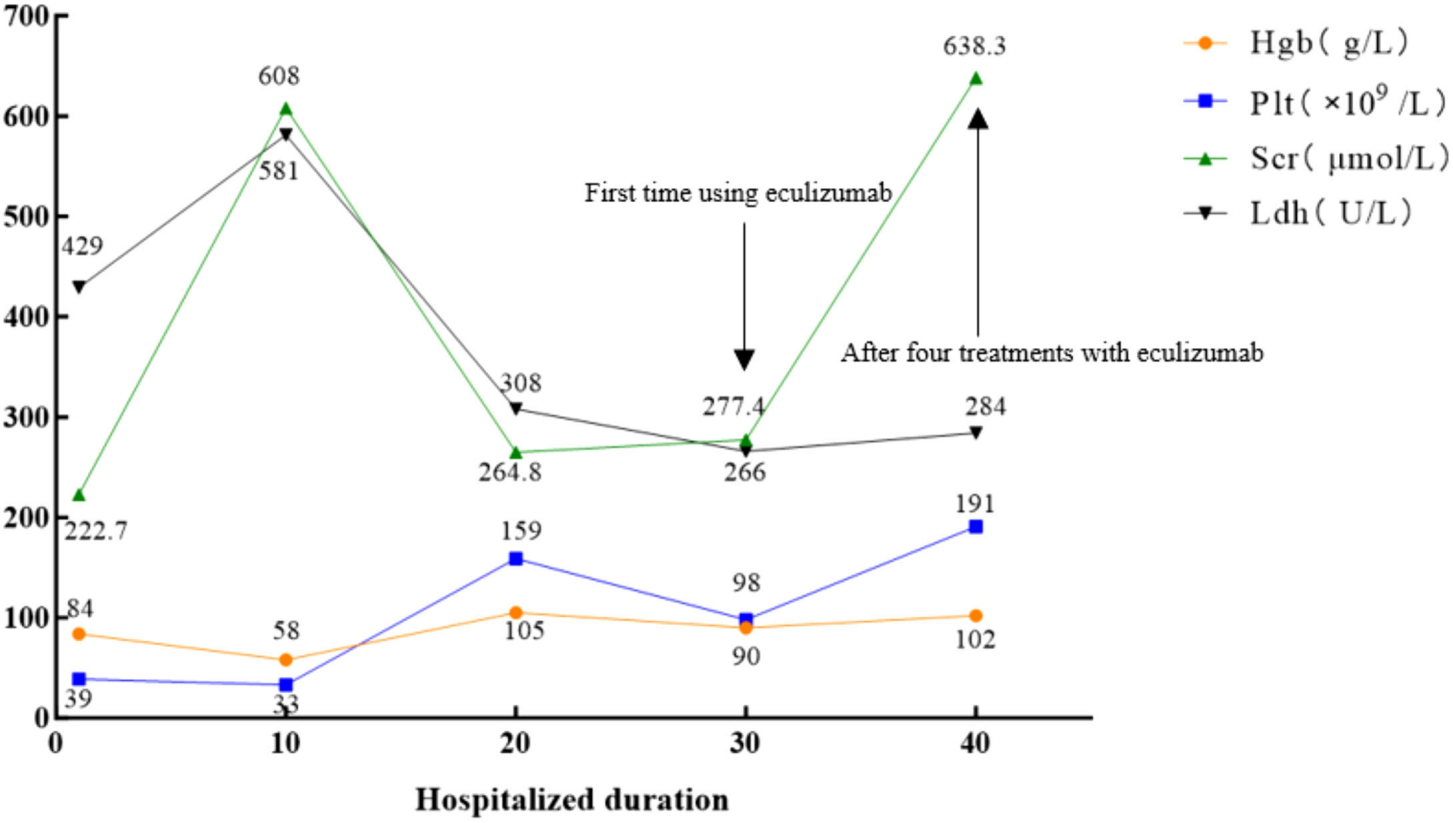
Biological parameters of the patient during the disease course. The normal values of Scr, Hgb, Plt, and Ldh are 88.4 μmol/L, 120 g/L, 100–300×10^9^/L, and 109–245 U/L, respectively. Hgb, hemoglobin; Plt, platelets; Scr, serum creatinine; Ldh, lactate dehydrogenase.

A 26-year-old Han Chinese female patient presented to the neurology department with intermittent seizures following an upper respiratory tract infection. Physical examination revealed symmetric pitting edema in both lower extremities with mild anemia. Brain magnetic resonance imaging (MRI) showed neurological abnormalities (**Figure 1**). In addition, cerebrospinal fluid biochemistry revealed elevated protein levels of 15.74 g/L and an increased white blood cell count of 204×10^6^/L in patients. The initial diagnosis was encephalitis, although examinations and diagnoses did not explain its etiology. Further auxiliary examinations revealed multiple abnormal clinical indicators suggesting hemolytic anemia and renal dysfunction (**Figure 2**). The patient’s lactate dehydrogenase level was 429 U/L, reticulocyte count was 8.26% (normal value: 0.59−2.07%), absolute reticulocyte count was 0.1768×10^12^/L (normal value: 0.0224−0.0829×10^12^/L), and immature reticulocyte fraction was 38.2% (normal value: 2.4−17.5%). Furthermore, plasma-free hemoglobin was 12 mg/L (normal value: <40 mg/L), and both the serum acidification hemolysis and direct anti-human globulin (IgG, C3d) tests were negative. The patient’s antinuclear antibody, antineutrophil cytoplasmic antibodies, and tumor marker tests were all negative. Urinalysis revealed urinary protein at 3+, urinary occult blood at 3+, and a haptoglobin level of <25 mg/dL(normal value: 30−200mg/dL) suggesting intravascular hemolysis. A bone marrow aspiration smear was performed to clarify the cause of the anemia, which indicated hyperplastic anemia with schistocytes. Blood cell morphology analysis revealed mild anisocytosis of mature red blood cells, with 1% schistocytes showing uneven distribution and visible platelet clumping.

Ten days after admission, the patient’s condition deteriorated rapidly, presenting with a deep coma, generalized edema, anuria, and malignant hypertension (blood pressure peaked at 240/130 mmHg). After careful inquiry during admission, there was no family history of TMA or related hematological disorders. The concurrent worsening of anemia, thrombocytopenia, and nervous system and kidney injuries (**Figure 2**) led to the suspicion of TMA, prompting emergency plasma exchange and hemofiltration. The patient’s overall condition improved following treatment (**Figure 2**); nevertheless, the patient was transferred to the nephrology department for further care owing to persistent anuria.

Considering the presentation of neuropsychiatric prodrome in adult female patients, TTP cannot be excluded. Laboratory tests showed 93% ADAMTS13 activity (<10% strongly suggests TTP), and the ADAMTS13 antibody test was negative. In these results, a TTP diagnosis was excluded. Additionally, targeted next-generation sequencing of multiple pathogens in the cerebrospinal fluid and blood revealed no pathogenic infections, further excluding STEC-HUS. The patient’s complement factor H antibody test was negative, with a complement factor H concentration of 233 μg/mL. Furthermore, the levels of complement C3 and C4 were normal, and both serum and urine immunofixation electrophoresis results were negative. From the follow-up brain MRI, a high likelihood of posterior reversible encephalopathy syndrome (PRES) was suggested (**Figure 1**). Considering all clinical and laboratory results, the final diagnosis was aHUS.

**Figure 3:**
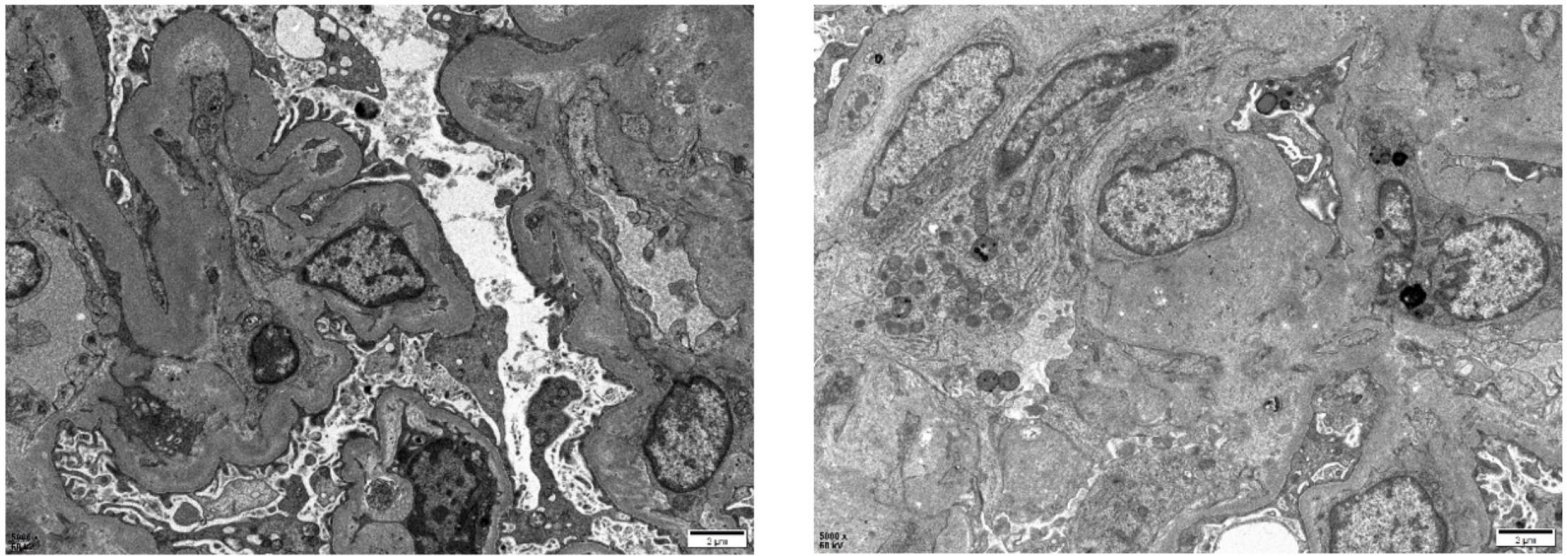
Pathological electron microscopic images of the patient’s kidney biopsy showed mild segmental hyperplasia of mesangial cells and stroma, diffuse contraction of the basement membrane with widening of the inner loose layer, and fusion of most epithelial foot processes. There was no evidence of definite electron-dense deposits. Other findings included vacuolar degeneration of renal tubular epithelial cells, increased number of lysosomes, partial exfoliation of microvilli, partial atrophy, and renal interstitial lymphomonocyte infiltration accompanied by collagen fiber hyperplasia.

**Figure 4:**
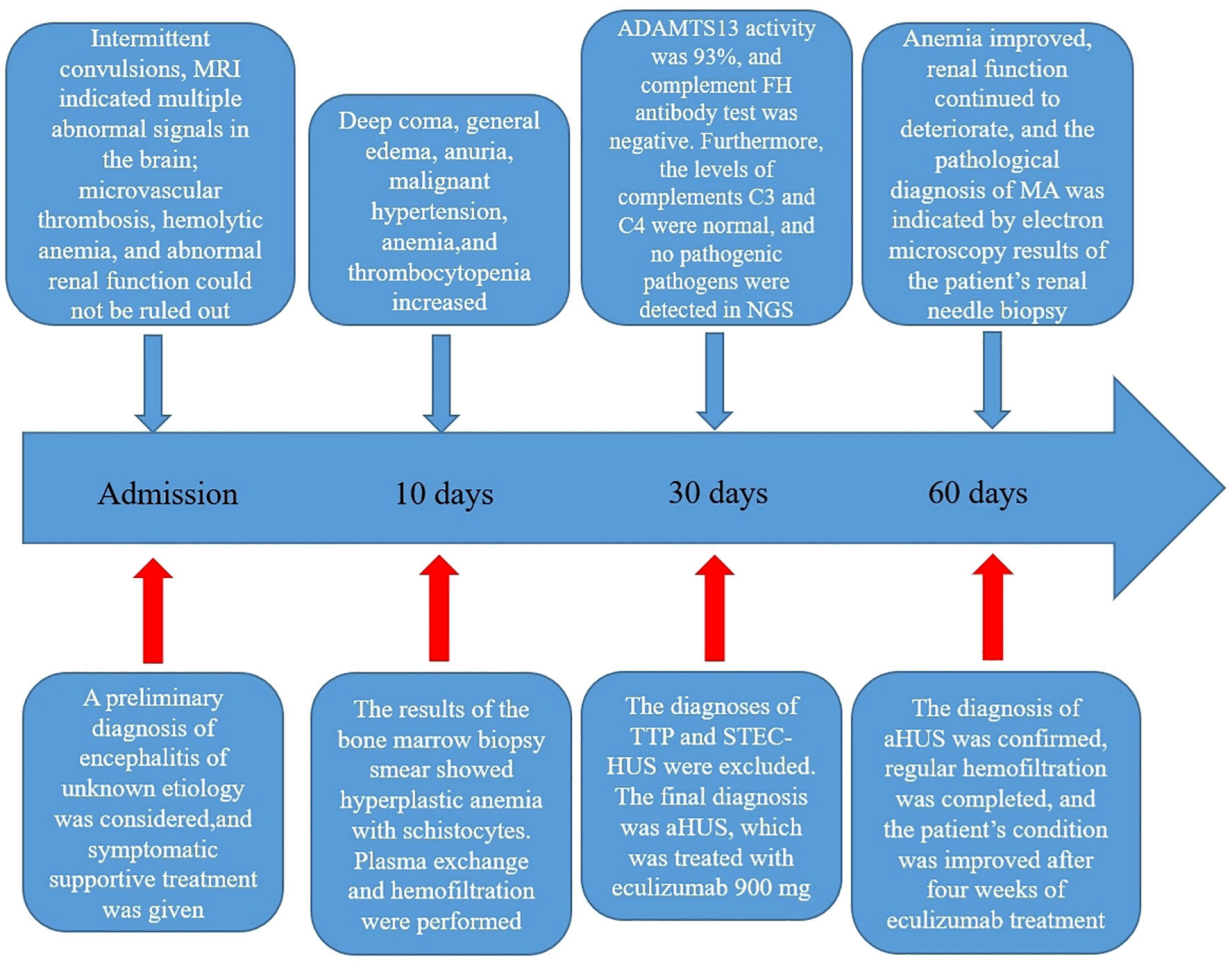
Steps involved in the diagnosis and treatment of the patient after admission. MRI, magnetic resonance imaging; NGS, next-generation sequencing; FH, factor H; TTP, thrombotic thrombocytopenic purpura; STEC-HUS, Shiga toxin-producing *Escherichia coli*-associated hemolytic uremic syndrome; aHUS, atypical hemolytic uremic syndrome; MA, microangiopathy.

Post-diagnostic treatment included eculizumab 900 mg weekly, plasma exchange, and intermittent hemodialysis. After the patient’s condition stabilized (**Figure 2**), a kidney biopsy was performed. Light microscopy revealed no clear glomeruli, vacuolar degeneration of renal tubular epithelial cells, protein casts in the tubules or other abnormalities. Electron microscopy (**Figure 3**) revealed glomerular microangiopathy, which supported the aHUS diagnosis. Following confirmation, regular hemofiltration was continued along with weekly 900 mg eculizumab for 4 weeks. After systematic treatment, anemia and hemolysis improved (**Figure 2**). A follow-up brain MRI showed improvement in PRES lesions, and neuropsychiatric symptoms did not recur (**Figure 1**), indicating effective treatment. The patient continued regular hemodialysis, with no clinical or laboratory evidence of disease recurrence (**Figure 4**).

## 3 Discussion

TMA is a group of clinicopathological syndromes characterized by microangiopathic hemolytic anemia, thrombotic thrombocytopenia, and ischemic organ damage caused by endothelial injury (3). aHUS, a type of TMA, is a rare and severe disease characterized by acute kidney injury, thrombocytopenia, and microangiopathic hemolytic anemia. Reports reveal an annual global incidence of approximately 1.5/million (4). Owing to its variable and nonspecific symptoms, rapid diagnosis of aHUS is challenging. Other conditions with similar presentations include STEC-HUS, TTP, and multisystem disease. Moreover, aHUS affects multiple systems, including the central nervous system (somnolence, seizures, encephalopathy, and cortical blindness), cardiovascular system (cardiomyopathy, myocardial infarction, and heart failure), pulmonary system (pulmonary hemorrhage), gastrointestinal system (pancreatitis and intestinal bleeding), and skeletal system (rhabdomyolysis), with such systemic complications occurring in up to 20% of cases (5).

According to the current classification, aHUS is not associated with infections or concurrent diseases. In some patients with aHUS, an infection (frequently in the upper respiratory tract) precedes the clinical TMA triad. Emerging evidence reveals that this phenomenon possibly reflects the role of infections and inflammatory states in exacerbating underlying complement pathway abnormalities in genetically susceptible individuals because systemic inflammation induces endothelial damage and propagates complement dysregulation (6, 7); thus, infection is typically considered a trigger rather than a cause (6). Instead, aHUS is usually associated with genetic defects in the regulation of complement activation in host cells and no known genetic defects and/or acquired inhibitors of complement proteins. Approximately 60% of patients with aHUS have genetic abnormalities (8, 9). Furthermore, 50–60% of patients have underlying genetic or acquired complement abnormalities (10). The pathogenesis of aHUS with genetic defects involves dysregulation of the complement cascade alternative pathway at the cell membrane level secondary to mutations in complement genes, which includes complement factor H (*CFH*), complement factor H-related 5 (*CFHR5*), complement factor I (*CFI*), and complement factor B (*CFB*), as well as complement component 3 (*C3*), CD46 (*MCP*), and thrombomodulin (*THBD*) (11). In contrast to genetic defects, acquired forms of aHUS may arise from autoantibodies targeting complement regulatory proteins, most notably the complement factor H antibody (12, 13). We tested our patient for relevant antibodies, and the results were negative. These findings effectively ruled out antibody-mediated complement dysregulation as a contributing etiology in this case. In addition, genetic testing was recommended to identify the mutated gene; however, the test was not performed because of the patient’s financial condition. Histologically, aHUS is a TMA, and its pathological features represent the tissue response to endothelial injury, typically showing intraluminal fibrin or fibrin-platelet plugs rather than thrombotic characteristics, such as endothelial swelling and desquamation, mesangiolysis, glomerular basement membrane double contours, and subendothelial accumulation of electron-lucent flocculent material (12). The results of our patient’s renal biopsy electron microscopy supported the diagnosis of aHUS, showing microangiopathy, which typically manifests in the kidney.

Owing to the variable and nonspecific symptoms of aHUS, no definitive diagnostic criteria exist, and clinical diagnosis relies on excluding similar diseases after the sudden onset of hemolytic anemia, thrombocytopenia, and acute kidney injury. Notably, patients with aHUS may present with neurological symptoms such as headache, confusion, seizures, and PRES (14). PRES is a reversible cortical-subcortical vasogenic brain edema condition that occurs with acute neurological symptoms (seizures, encephalopathy, headache, and visual disturbances) in the context of renal failure, blood pressure fluctuations, cytotoxic drugs, autoimmune diseases, or preeclampsia/eclampsia. It typically presents as vasogenic edema on brain imaging and predominantly affects the bilateral parieto-occipital regions (15). These symptoms may be relatively less common in aHUS than in other TMAs. In this case, the patient’s initial presentation of intermittent seizures and subsequent PRES was notably rare. In previous research, it was suggested that the likelihood of patients presenting with neurological signs and symptoms varies by the TMA type, and current findings indicate a similar probability in TTP and HUS. Some patients experience seizures and neuroimaging abnormalities; nevertheless, such abnormalities are possibly less common in aHUS (16), which complicated our initial assessment and association of the patient’s intermittent seizures and abnormal cranial neuroimaging with aHUS. Kenjale et al. (17) reported the case of a 15-year-old Indian boy diagnosed with aHUS who presented with a generalized tonic-clonic seizure on the morning of admission as well as anemia, papilledema, hypertension, paroxysmal epilepsy, and confirmed PRES. Chan and Weinstein (18) reported a case of a 14-month-old unimmunized girl who was diagnosed with aHUS after presenting with neurological symptoms (seizures and nystagmus). Mostafa et al. (19) also reported the case of a 21-year-old woman diagnosed with aHUS after presenting with partial seizures, hypertension, acute renal failure, and severe thrombocytopenia. The diagnostic criteria for aHUS include microangiopathic hemolytic anemia, consumptive thrombocytopenia, and organ damage due to microcirculatory thrombosis. After confirming aHUS, complementary serological testing is useful in subsequent disease follow-up and monitoring.

Recently, our understanding of aHUS treatment has increased. Typical aHUS is primarily related to mutations or autoantibodies that cause dysregulation of complement activation, and in most aHUS cases, this process can be halted through therapeutic complement inhibition (6). Eculizumab is a monoclonal antibody that blocks C5 cleavage, thereby preventing the formation of the pro-inflammatory peptides C5a and C5b-9 membrane attack complex, which are central to the pathophysiology of aHUS (20). Findings from an increasing number of clinical studies and case reports on patients with varying histories have revealed the effectiveness of eculizumab in preventing further deterioration in patients with aHUS (21). Regarding long-term prognosis, findings from a prospective non-randomized trial showed hematologic remission with normalization of platelet count within 7–8 days and LDH level after 14–54 days. For kidney function recovery, a mean estimated glomerular filtration rate recovery of 64 mL/min/1.73m^2^ in children and 30–35 mL/min/1.73m^2^ in adults was observed (20). Our patient showed good hematological and neurological responses but, unfortunately, remained dialysis-dependent despite the use of eculizumab. This may have been due to delayed initiation of eculizumab treatment. The critical importance of early complement blockade has been emphasized in molecular studies highlighting irreversible C5b-9-mediated podocyte detachment within 6–12 h of uncontrolled complement activation (22). Earlier intervention using eculizumab was associated with significantly greater improvement in the estimated glomerular filtration rate, improved clinical outcomes, and reversal of organ damage (20). Walle et al. (23), in their retrospective analysis, further validated that renal outcomes improved when eculizumab was started within 7 days of diagnosis.

Regarding therapeutic optimization, emerging evidence supports using ravulizumab—a long-acting humanized monoclonal anti-C5 antibody engineered through minimal structural modifications of eculizumab (24). While maintaining equivalent C5-binding affinity and identical terminal complement blockade mechanisms, ravulizumab exhibits an extended elimination half-life (∼52 days vs. ∼11 days with eculizumab), which permits 8-weekly maintenance dosing after the initial loading (25). Notably, in recent clinical observations, ravulizumab’s efficacy has been demonstrated across complex scenarios. Matošević et al. (26) reported successful complement inhibition in a patient with COVID-19-associated aHUS. These findings suggest potential therapeutic advantages in hyperinflammatory states where accelerated complement consumption may compromise eculizumab efficacy. For our dialysis-dependent patient—currently stabilized on biweekly eculizumab—transition to ravulizumab could mitigate cumulative treatment burden while maintaining target C5 inhibition (>99% suppression at trough concentrations ≥50 μg/mL) (27). Meanwhile, pharmacoeconomic analyses indicate annual cost parity between the two agents (28).

Genetic testing also has an impact on the choice of treatment. In cohort studies, patients with mutations in the *MCP* gene presented a significantly earlier onset of the disease than those with *CFH*, *CFI*, or no identified mutation. *CFH* mutations were associated with a significantly worse renal prognosis in adult patients who had poorer renal outcomes (29). If genetic test results are not available (as was observed in our case), the recommendations of current guidelines involve vigilant monitoring for hematuria and proteinuria, with immediate re-initiation of anti-C5 therapy upon relapse (30).

In addition, new questions have emerged in recent years regarding early and long-term plasma exchange in the treatment of aHUS. Owing to the lack of definitive diagnostic tests for aHUS, plasma exchange is often used as the first-line HUS treatment before further disease evaluation, despite the risk of complications and limited evidence. In a recent study, the feasibility of treating severe TMA renal injury with C5 complement inhibitors without plasma exchange was explored. The study’s conclusion was that early C5 complement inhibitor treatment without plasma exchange showed promising initial results in patients with suspected aHUS with severe TMA renal injury (31). Regarding plasma exchange treatment after confirming aHUS, it was revealed in the long-term follow-up that 78% of patients died or developed end-stage renal disease with extended plasma exchange treatment (32). Therefore, early plasma exchange in the treatment of aHUS remains controversial and should not be used indefinitely.

In conclusion, aHUS should be considered in patients presenting with neurological symptoms accompanied by acute kidney injury, thrombocytopenia, and microangiopathic hemolytic anemia. Once diagnosed, early eculizumab administration is crucial for improved disease control and kidney function protection. Additional information on neurological symptoms and specific imaging or indicator changes in patients with aHUS will further clarify the early symptoms of this rare disease, aiding in earlier diagnosis and appropriate treatment implementation.

## Data Availability

The original contributions presented in the study are included in the article/supplementary material. Further inquiries can be directed to the corresponding authors.
